# In Silico Study to Enhance Delivery Efficiency of Charged Nanoscale Nasal Spray Aerosols to the Olfactory Region Using External Magnetic Fields

**DOI:** 10.3390/bioengineering9010040

**Published:** 2022-01-16

**Authors:** Benjamin Li, Yu Feng

**Affiliations:** 1 Research in Computational Science, North Carolina School of Science and Mathematics, Durham, NC 27705, USA; benli20042013@gmail.com; 2 School of Chemical Engineering, Oklahoma State University, Stillwater, OK 74078, USA

**Keywords:** nasal drug delivery, nose-to-brain drug delivery, computational fluid dynamics

## Abstract

Various factors and challenges are involved in efficiently delivering drugs using nasal sprays to the olfactory region to treat central nervous system diseases. In this study, computational fluid dynamics was used to simulate nasal drug delivery to (1) examine effects on drug deposition when various external magnetic fields are applied to charged particles, (2) comprehensively study effects of multiple parameters (i.e., particle aerodynamic diameter; injection velocity magnitude, angle, and position; magnetic force strength and direction), and (3) determine how to achieve the optimal delivery efficiency to the olfactory epithelium. The Reynolds-averaged Navier–Stokes equations governed airflow, with a realistic inhalation waveform implemented at the nostrils. Particle trajectories were modeled using the one-way coupled Euler–Lagrange model. A current-carrying wire generated a magnetic field to apply force on charged particles and direct them to the olfactory region. Once drug particles reached the olfactory region, their diffusion through mucus to the epithelium was calculated analytically. Particle aerodynamic diameter, injection position, and magnetic field strength were found to be interconnected in their effects on delivery efficiency. Specific combinations of these parameters achieved over 65-fold higher drug delivery efficiency compared with uniform injections with no magnetic fields. The insight gained suggests how to integrate these factors to achieve the optimal efficiency.

## 1. Introduction

Central nervous system (CNS) disorders, which include neurological disorders, are a major category of disorders worldwide. In 2019, neurological disorders resulted in 533,172 deaths among 33 countries in North and South America, as well as 7.5 million total years of life lost [1]. Neurological disorders also caused 12% of global deaths in 2006 [2]. Examples include Alzheimer’s disease, Parkinson’s disease, and brain cancer, such as glioblastoma multiforme, which affects over 10,000 people annually and has a typical survival time of fewer than two years [3].

With intravenous drug delivery to treat CNS disorders, an obstacle exists, i.e., the blood–brain barrier (BBB). The BBB consists of an endothelial cell layer around the vasculature within the brain. Tight junctions exist between these endothelial cells, which limit drug permeability through BBB in order to reach the target regions of the brain. The BBB restricts nearly all large molecule drugs and 98% of small molecule drugs [4]. Nasal drug delivery offers a pathway to avoid BBB. By delivering drugs to the olfactory region at the top of the nasal cavity, drug molecules can pass through the olfactory epithelium to pathways leading to brain tissue. This is known as nose-to-brain drug delivery [5].

Efficiently transporting drugs to the olfactory region is also a challenge that has been studied. Multiple modes of drug delivery in the nasal cavity exist, including dry powder and liquid aerosol sprays. Inhaled nasal sprays, which inject particles or droplets on the scale of nanometers or microns, are commonly used to administer drugs in a non-invasive way, as particles can adhere to the mucus that lines the interior of the nasal cavity [6]. However, when nasal sprays have been tested on their own, low delivery efficiency has been achieved. Previous studies often found low deposition rates, with most of the drug depositing in other areas of the nasal cavity or exiting the nasal cavity [7,8].

Computational fluid dynamics (CFD) has been proved to be suitable to simulate the process of nasal drug delivery, which is advantageous to test many different configurations of drug delivery systems and accurately determine quantitative information such as airflow velocity and pressure, as well as particle tracks [8,9,10,11,12,13,14,15,16]. Specifically, CFD has been used to realistically simulate nasal airflow. A computational mesh of the airway can be generated, and airflow can be resolved within the domain. Multiple Reynolds-averaged Navier–Stokes (RANS) turbulence models have been used to predict the laminar-to-turbulent transitional airflow field in the nasal cavity. Those RANS models include two-equation models such as the k-ϵ and k-ω models. Zhang and Kleinstreuer [9] compared multiple RANS models with large eddy simulation (LES) for simulations of oral airflow, which contains transitions from laminar to turbulence. They found that there are no significant differences between the low Reynolds number k-ω model, transition shear–stress transport (SST) model, and LES model. Their study also concluded that the transition SST model provides good predictions of turbulent kinetic energy distributions. Furthermore, this study also compared predicted velocities calculated using the above-mentioned turbulence models with experimental results. Similarly, Inthavong et al. [10] also employed the transition SST model for nasal airflow predictions. Thus, based on the existing benchmark studies mentioned above, the transition SST model can be employed to provide accurate airflow predictions in the nasal cavity, especially for the predictions of transition sites between laminar and turbulence.

CFD modeling of the airflow can be coupled with a Lagrangian particle tracking model, i.e., the discrete phase model (DPM), to simulate the motion of drug particles within the nasal airflow. Inthavong et al. [11] used a one-way coupled DPM to simulate the transport and delivery of therapeutic microparticles via nasal spray in the nasal cavity. This study examined particle deposition for varying particle diameters from 1 μm to 80 μm with a constant inhalation air flow rate of 15 L/min. They found that their results, specifically the deposition efficiency in the nasal cavity based on an inertial parameter, were comparable with experimental measurements.

There are several recent studies that have investigated various approaches to increase drug delivery efficiency to the olfactory region. One technique is to use a controlled drug release method based on the particle release map, as done by Vachhani and Kleinstreuer [12]. In this method, particle trajectories are tracked, and starting positions of particles reaching the olfactory region are found, giving favorable locations at the nasal inlet for targeted nasal spray injections. The study modeled drug delivery for particles with diameters from 1 nm to 500 nm and airflow rates from 5 L/min to 20 L/min. It was found that using this targeted injection technique resulted in significantly increased drug delivery efficiency compared with injecting the drug uniformly across the nasal inlet. For instance, 1 nm particles reached 53.207% delivery efficiency when the particle release map was used for a targeted injection, while only 3.728% delivery efficiency was achieved for a uniform injection.

Another method to increase olfactory delivery efficiency is magnetic drug targeting [8,13,17,18,19,20]. Specifically, externally generated magnetic or electric fields have been used to guide magnetic or charged drug particles to designated delivery sites, not only in airways [18,19] but also in blood vessels [20]. Focusing on the targeted delivery in nasal cavities, Xi et al. [17] proposed a magnetophoretic olfactory delivery device design and found that the device can increase the olfactory delivery efficiency by 1.5-fold compared with the baseline drug delivery strategy without using the device. Following this study, Xi and Si [8] developed computational and experimental models to study olfactory drug delivery, using electrodes to attract charged drug particles towards the front of the nasal cavity to improve olfactory deposition. When electric fields were applied, olfactory deposition increased by a factor of 5.2 compared with the deposition in the absence of external fields. In addition to electrodes being used to generate electric fields, a current-carrying wire can be used to create a magnetic field to guide drug particles. Pourmehran et al. [13] applied a current-carrying wire to control the trajectories of particles in the lung and enhance the deposition efficiency at designated locations.

Once drug particles reach the olfactory region, the drug should diffuse through the mucus layer before reaching the olfactory epithelium, where the drug can be absorbed. Such drug diffusion processes through the mucus have been modeled mathematically. Shang et al. [14] used CFD and modeled drug deposition and mucociliary clearance in the nasal cavity. They used the analytical solution for a drug diffusion model derived by Erickson et al. [21].

Other factors that can influence drug delivery efficiency include the particle diameter, injection angle, inhalation flow rate, injection velocity magnitude, and acoustic wave [15,16,22]. When targeted injection and external fields are introduced, those parameters mentioned above must be also optimized. Existing studies have examined the effects of individual or several parameters, but there is not a sufficient understanding of the integration of all these parameters. Additionally, many studies consider microparticle nasal sprays, but more research is needed on nanoparticle delivery, especially in integrating nanoparticle drug delivery with the previously mentioned factors. Additionally, studies have examined the effects of external magnetic fields, but more work is needed on specific magnetic field strengths that are most suitable for particular sizes of particles and configurations of other parameters.

The goal of this study is to simulate nasal drug delivery using a previously validated one-way coupled Euler–Lagrange model (i.e., the one-way DPM) [23,24], while varying particle the aerodynamic diameter; the velocity magnitude, angle, and position of nasal spray injection; and the magnetic field strength to determine their effects on olfactory delivery efficiency. Accordingly, this study aims to identify configurations of parameters that lead to enhanced efficiency. As a potential factor that can influence the targeted delivery efficiency, the acoustic wave [22] was not considered in this study. RANS equations were used to model nasal airflow, and the transition SST model [25,26] was applied to resolve the laminar-to-turbulence transition. Additionally, targeted injections were applied based on data collected from uniform injections and the particle release map technique [12]. The magnetic field is generated by a current-carrying wire near the front of the nasal cavity, to allow for the testing of configurations to predict delivery efficiency. Potential novel contributions of this study include (1) the investigation of how integrating the targeted injection strategy and external magnetic field control can enhance the delivery efficiency of drug particles to the olfactory region, and (2) the prediction of subsequent drug diffusion through the mucus at the olfactory region using an analytical solution. Insight from this study informs future work by suggesting connections between important factors of nasal drug delivery systems to consider in the integration of such factors to achieve the best efficiency.

## 2. Materials and Methods

### 2.1. Geometry and Mesh

**Figure 1:**
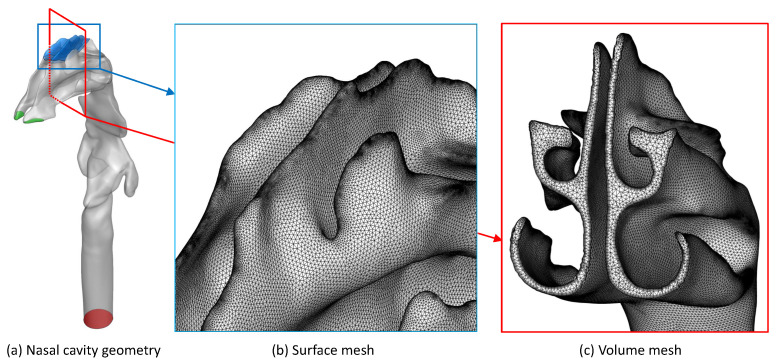
Details of the nasal airway model: (**a**) Geometry, (**b**) Surface mesh, and (**c**) Volume mesh.

Figure 1 displays the nasal airway geometry and CFD mesh. This computer-aided design (CAD) geometry represents the nasal passage of a healthy 53 year old, non-smoking male [27].

The blue region in Figure 1a indicates the olfactory region, which is the target location for drug delivery in this study. In green are the nasal inlets, or nostrils, where airflow and nasal sprays enter the nasal cavity. The red region indicates the outlet at the base of the trachea, where airflow exits.

Four finite volume meshes were generated using Ansys Fluent Meshing 2020 R2 (Ansys Inc., Canonsburg, PA, USA). Meshes consisted of tetrahedral cells making up the volume mesh, as well as five layers of prism cells near the nasal cavity walls. Figure 1b and Figure 1c display the surface and volume mesh for the mesh with 4.4 million cells.

A mesh independence test was conducted on the four meshes. Nasal airflow was resolved using the transition SST model. The mass flow rate at each nasal inlet was set to be constant at 0.0002042 kg/s. Four meshes were generated with different levels of refinement for the mesh independence test, with 1, 2, 4.4, and 8 million cells. Velocity magnitude and pressure were recorded at 20 selected monitor points, approximately evenly spaced through the fluid domain. Percentage differences from more refined meshes to less refined ones were calculated. Among the 20 monitor points distributed through the computational domain, the greatest velocity difference between meshes with 4.4 million and 8 million cells was 10.45%, and the greatest pressure difference was 9.21%. At 11 of 20 points, pressure and velocity differed by less than 2%, and these differed by less than 5% at 16 of 20 points. The average velocity and pressure differences over all 20 points were 2.55% and 1.56%, respectively. Between the meshes with 4.4 million and 8 million cells, differences in fluid flow were not significant. Therefore, to achieve the optimal balance between computational efficiency and accuracy, the mesh with 4.4 million cells was selected for the simulations in this study.

### 2.2. Governing Equations

#### 2.2.1. Continuous Phase

To model the laminar-to-turbulence transitional airflow in the nasal cavity geometry, the RANS equations were employed and given as follows [28]:

(1a) $$\begin{array}{cc} \frac{\partial \rho }{\partial t}+\frac{\partial }{\partial x_{i}}\left(\rho u_{i}\right) & =0 \end{array}$$

(1b) $$\begin{array}{cc} \frac{\partial }{\partial t}\left(\rho u_{i}\right)+\frac{\partial }{\partial x_{j}}\left(\rho u_{i}u_{j}\right) & =-\frac{\partial p}{\partial x_{i}}+\frac{\partial }{\partial x_{j}}\left[\mu \left(\frac{\partial u_{i}}{\partial x_{j}}+\frac{\partial u_{j}}{\partial x_{i}}-\frac{2}{3}\delta_{ij}\frac{\partial u_{l}}{\partial x_{l}}\right)\right]+\frac{\partial }{\partial x_{j}}\left(-\rho \bar{u_{i}^{\prime }u_{j}^{\prime }}\right) \end{array}$$

where *t* is time, *u* is velocity, ρ is the air density, *p* is pressure, μ is the air viscosity, and $-\rho \bar{u_{i}^{\prime }u_{j}^{\prime }}$ is the Reynolds stress. To resolve the Reynolds stress and close the equation system, the validated transition SST model [25,26] was employed. The transition SST model consists of four equations and is based on the widely used k−ω SST model, but it integrates two more transport equations for intermittency (γ) and transition momentum thickness Reynolds number (${\tilde{Re}}_{\theta t}$).

#### 2.2.2. Discrete Phase

Trajectories of drug particles carried by the airflow were modeled by a one-way coupled Lagrangian DPM, because the particle volume fraction in the computational domain is much lower than 10%. Trajectories were influenced by multiple forces, such as drag, gravity, and magnetic force. Newton’s Second Law was employed to describe the force balance on each particle [29,30], i.e.,

(2) $$m_{p}\frac{d{\vec{u}}_{p}}{dt}={\vec{F}}_{m}+{\vec{F}}_{D}+{\vec{F}}_{VM}+{\vec{F}}_{PG}+{\vec{F}}_{Gravity}$$

where $m_{p}$ is particle mass and ${\vec{u}}_{p}$ is particle velocity. ${\vec{F}}_{D}$ represents the drag force on nanoparticles, which is defined by the Stokes–Cunningham drag law [31]. Specifically, ${\vec{F}}_{D}$ can be calculated by

(3) $${\vec{F}}_{D}=m_{p}\frac{18\mu }{d_{p}^{2}\rho_{p}C_{c}}\left(\vec{u}-{\vec{u}}_{p}\right)$$

where $\vec{u}$ is the airflow velocity, $C_{c}$ is the Cunningham correction factor, and $d_{p}$ is the particle aerodynamic diameter. ${\vec{F}}_{Gravity}$ in Equation (2) represents the gravitational force. The remaining terms represent additional forces exerted on a particle, i.e., magnetic force, Saffman’s lift force, virtual mass force, and pressure gradient force. Equation (4) describes the magnetic force ${\vec{F}}_{m}$ exerted by the magnetic field due to the current-carrying wire, i.e.,

(4) $${\vec{F}}_{m}=q{\vec{u}}_{p}\times \vec{B}$$

where *q* is particle charge and $\vec{B}$ is magnetic field. Expressions of other forces considered in Equation (2), i.e., Saffman’s lift force ${\vec{F}}_{Saffman}$ [32,33], virtual mass force ${\vec{F}}_{VM}$ and pressure gradient force ${\vec{F}}_{PG}$, are defined by the following equations [28]:

(5a) $$\begin{array}{cc} {\vec{F}}_{Saffman} & =\frac{2Kv^{0.5}\rho d_{ij}}{\rho_{p}d_{p}{\left(d_{lk}d_{kl}\right)}^{0.25}}\left(\vec{u}-\vec{u_{p}}\right) \end{array}$$

(5b) $$\begin{array}{cc} {\vec{F}}_{VM} & =\frac{1}{2}m_{p}\frac{\rho }{\rho_{p}}\frac{d}{dt}\left(\vec{u}-{\vec{u}}_{p}\right) \end{array}$$

(5c) $$\begin{array}{cc} {\vec{F}}_{PG} & =m_{p}\frac{\rho }{\rho_{p}}{\vec{u}}_{p}\cdot \nabla \vec{u} \end{array}$$

Since the density difference between airflow and particles is high, the virtual mass force ${\vec{F}}_{VM}$ is negligible. In addition, since the particle density is much higher than the airflow density, the pressure gradient force ${\vec{F}}_{PG}$ is also negligible. To determine the interaction between the particles as the discrete phase and the airflow as the continuous phase, a one-way coupled Euler–Lagrangian approach was used. Only particle motion was influenced by airflow, while the effects on airflow due to the presence of the discrete phase were neglected because of the low particle volume and mass fractions in the computational domain.

#### 2.2.3. Boundary Conditions

All inner walls of the nasal airway were no-slip boundaries. The discrete phase boundary condition was set to trap all particles contacting the inner walls. The position, time of deposition, region, particle size, injection ID, and particle ID were tracked for all deposited particles using a customized user-defined function (UDF).

Olfactory deposition efficiency, referred to simply as deposition efficiency, was measured by the ratio of the number of particles deposited in the olfactory region to the total number of particles per injection.

Both nasal inlets had mass flow rates normal to the boundary face. Intermittency was set to 1.0, turbulent intensity at 2.7% [34], and turbulent viscosity ratio at 10.0. A realistic breathing waveform was applied as the transient nasal inlet condition. The breathing waveform is defined by the sinusoidal series given in Equation (6) [35], with coefficients displayed in Table 1. Airflow was simulated from *t* = 0.20 s to *t* = 0.70 s.

(6) $${\dot{m}}_{in}=\sum_{i=1}^{7}a_{i}\sin\left(b_{i}\left(t-0.2\right)+c_{i}\right)$$

#### 2.2.4. Initial Conditions

Particles in each injection were assumed to contain particles of equal diameter and charge, as well as uniform injection velocity. Particles were assumed to be spherical water droplets.

Uniform and targeted release injections were simulated. Uniform injections consisted of 8935 particles evenly distributed about both nasal inlets, at a plane 1 mm deep into the inlets. Based on the approximate starting positions of particles in uniform injections reaching the olfactory region, release positions were selected.

**Figure 2:**
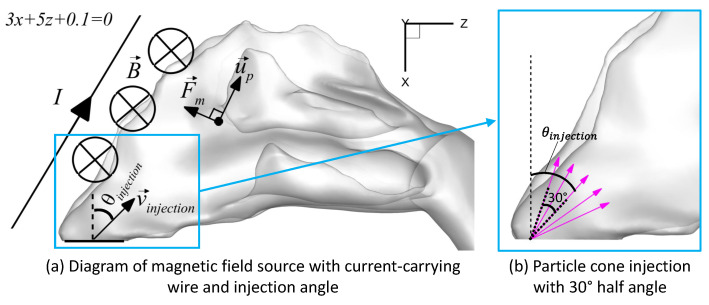
Magnetic field source and spray cone: (**a**) Diagram of magnetic field source with current-carrying wire and injection angle, and (**b**) Velocity vectors of particles injected by a spray cone.

Targeted injections consisted of 5000 particles injected from specific positions 1 mm deep into the inlets, totaling 10,000 particles. A cone injection was used for targeted release, with a constant spray cone half angle of $30^{\circ }$ (see Figure 2b). The injection angle was measured from the normal vector to the nasal inlets (i.e., the vertical axis) to the axis of the cone. An initial targeted injection position was tested, and this position was also adjusted by moving it back along the nasal inlets.

For both uniform and targeted release injections tested, parameters of nasal sprays were the particle aerodynamic diameter (1 nm ≤ *d*_*p*_ ≤ 1000 nm), injection velocity magnitude (0.5 m/s ≤ *u*_*injection*_ ≤ 25 m/s), and injection angle (−60° ≤ *θ*_*injection*_ ≤ 75°). The injection angle was the angle in the xz plane between the normal vector of the nasal inlets and the injection velocity vector (see Figure 2a).

#### 2.2.5. External Magnetic Field

The magnetic targeting system was defined by an infinite, linear, current-carrying wire represented by the line in Equation (7), which is also displayed in Figure 2a.

(7) 3x+5z+0.1=0

The current traveled in the negative *x* direction. At the xy plane, the magnetic field $\vec{B}$ induced by the current in the wire was in the positive *y* direction, and its magnitude can be calculated by

(8) $$\left|\right|\vec{B}\left|\right|=\frac{I\mu_{0}}{2\pi d}$$

where *d* is the distance from a particle to the wire, $\mu_{0}$ is the permeability of free space, and *I* is the current. The magnetic force acting on the charged particles can be calculated by Equation (4).

The current through the wire was modulated from 0 A to 100,000 A. The charge *q* on particles was assumed to be proportional to particle volume, similar to the correlation in the study of Golshahi et al. [36]. A cubic relationship between the particle charge and particle aerodynamic diameter was assumed, with particles with aerodynamic diameter 1 nm having a charge of 1 electron, i.e.,

(9) $$q=\frac{\pi }{6}{\left(d_{p}\right)}^{3}\rho_{e}$$

where $d_{p}$ is the particle aerodynamic diameter, $\rho_{e}$ is the particle charge density, and *e* is the charge magnitude of an electron. Specifically, $\rho_{e}$ can be given as

(10) $$\rho_{e}=\frac{1\;e}{{\left(1\;\mathrm{nm}\right)}^{3}\pi /6}$$

Thus, the magnetic force exerted on a particle was proportional to particle mass. Regardless of size, particles experienced the same magnetic force per mass.

### 2.3. Analytical Solution for Drug Diffusion through the Mucus

Equation (11a)–(11c) defined drug particle diffusion through the olfactory mucus layer. They represent the diffusion equation, boundary conditions, and initial conditions [21]:

(11a) $$\begin{array}{cc} \frac{\partial u}{\partial t} & =D_{m}\frac{\partial^{2}u}{\partial x^{2}} \end{array}$$

(11b) $$\begin{array}{cc} \frac{\partial u(0,t)}{\partial x}=0 & ,u(L,t)=0 \end{array}$$

(11c) $$\begin{array}{cc} u(x,0) & =2\cdot \delta (x) \end{array}$$

where u(x,t) is the drug concentration at a depth of *x* in the mucus at a time of *t*, *L* is the height of the mucus layer, x∈[0,L], $D_{m}$ is the diffusion coefficient of the drug in mucus, and δ(x) is the Dirac function. The analytical solution of this mucus diffusion model in Equation (12a)–(12d) was used to model drug diffusion:

(12a) $$\begin{array}{cc} u(x,t) & =\frac{2}{L}\sum_{n=1}^{\infty }e^{\lambda_{n}^{2}t}\cos\left(\frac{2n-1}{2L}\pi x\right) \end{array}$$

(12b) $$\begin{array}{cc} \lambda_{n}^{2} & ={\left(\frac{2n-1}{2L}\pi \right)}^{2}D_{m} \end{array}$$

(12c) $$\begin{array}{cc} D_{m} & =D_{w}e^{\frac{-\pi }{4}{\left(\frac{r_{s}+r_{f}}{r_{g}+r_{f}}\right)}^{2}} \end{array}$$

(12d) $$\begin{array}{cc} D_{w} & =\frac{k_{B}T}{6\pi \mu_{w}r_{s}} \end{array}$$

for n=1,2,3,… and x∈ [0, L]. *L* is the mucus thickness, assumed as 10 μm [37], $D_{w}$ is the drug diffusivity in water, $r_{g}$ is the mucin network’s effective mesh fiber spacing of 50 nm, $r_{f}$ = 3.5 nm is the mucin fiber radius [38], $r_{s}$ is the effective solute radius, $k_{B}$ is the Boltzmann constant, and $\mu_{w}$ is water viscosity. It is worth mentioning that Equation (12c) uses the obstruction-scaling model [39], and Equation (12d) uses the Stokes–Einstein equation [40]. This analytical solution was evaluated and plotted using MATLAB R2021a (MathWorks, Natick, MA, USA).

### 2.4. Numerical Setup

CFD simulations were performed using Ansys Fluent 2020 R2 (Ansys Inc., Canonsburg, PA, USA) and were run on the TIGER Research Cloud (8 Intel Core Processors (Haswell, IBRS) 2.29 GHz with 64 GB RAM) at Oklahoma State University (OSU) High Performance Computing Center (HPCC). Each case took approximately 14 h to compute airflow and particle trajectories for 500 flow time steps of length 0.001 s. The SIMPLE scheme was used for pressure–velocity coupling and the least-squares cell-based scheme for the discretization of cell gradient. The second-order scheme was applied for the discretization of pressure, and the second-order upwind scheme was applied for the discretization of momentum, turbulent kinetic energy, specific dissipation rate, intermittency, and momentum thickness Re. Convergence was defined when residuals were less than $10^{-3}$.

### 2.5. Model Validation

The transition SST model has been extensively validated and employed in previous research to resolve the flow field based on its ability to predict pressure drop and velocity profiles accurately and to predict shear stress for both transitional and turbulent flows in airways [23,24]. The one-way coupled Euler–Lagrange method was also well-proved with in vitro and in vivo data in the previous research for accurate predictions of the aerosol dynamics in human respiratory systems [24,41].

## 3. Results

### 3.1. Airflow in the Nasal Cavity

**Figure 3:**
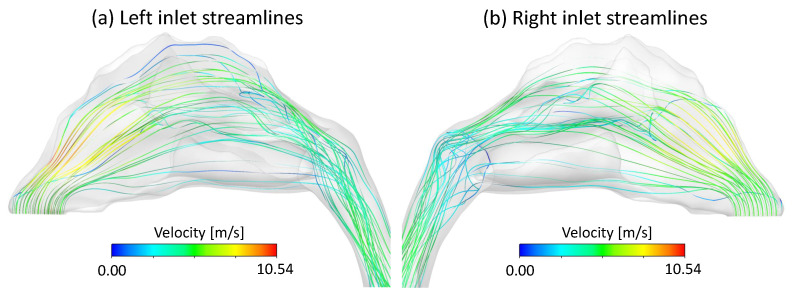
Streamlines of airflow velocity: (**a**) Left inlet streamlines, and (**b**) Right inlet streamlines.

**Figure 4:**
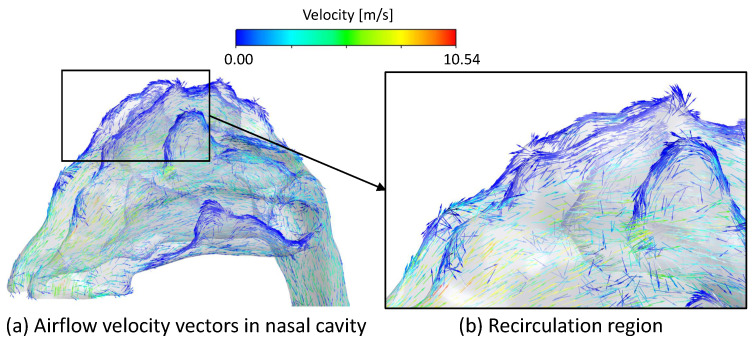
Velocity vectors in the nasal cavity in front of the olfactory region: (**a**) Airflow velocity vectors in nasal cavity, and (**b**) Recirculation region.

Figure 3 displays streamlines of airflow entering the left and right nasal inlets, colored by the local velocity magnitude. On both sides, air entering at the anterior or middle region of the inlet increased in velocity as it passed the angled part of the nasal cavity’s front wall. The airflow then decreased in velocity traveling near the olfactory region and the central regions of the nasal cavity, respectively. Air entering from the posterior of the nasal inlet flowed along the floor of the nasal cavity and decreased in velocity. Only a few streamlines reached the upper boundary of the nasal cavity. Figure 4a displays airflow velocity vectors in the left side of the nasal cavity, and Figure 4b focuses on the upper regions of the left side of the nasal cavity near the anterior of the olfactory region, where there was also a region of slow, recirculating air, displayed by the airflow vectors traveling in the direction back toward the nasal inlets.

### 3.2. Particle Deposition: Uniform Injections

**Figure 5:**
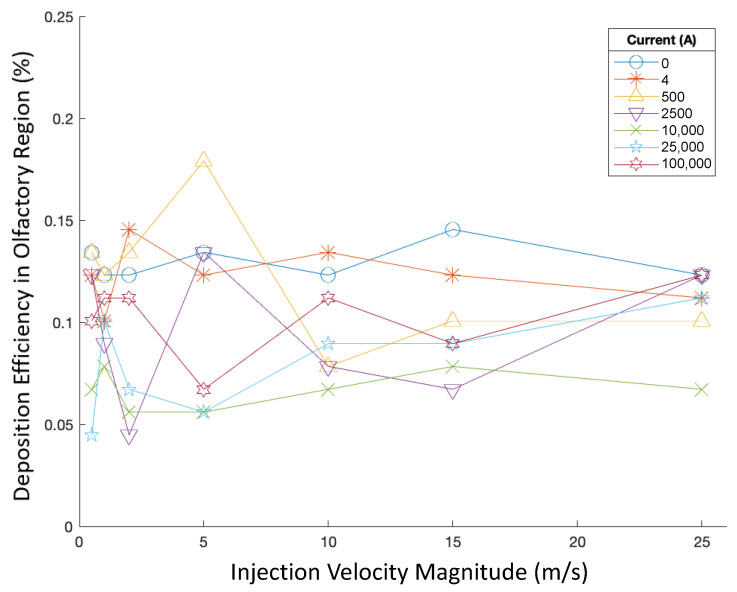
Deposition efficiencies in varying magnetic fields for varying injection velocities.

The deposition efficiency (DE) in the olfactory region with different parameter values is visualized and compared in this section in order to find the optimized injection strategy to achieve the highest DE. Specifically, olfactory deposition was first recorded for varied injection velocities and currents. Particles were injected uniformly across the nasal inlets perpendicular to the inlet boundaries. Particles had a constant aerodynamic diameter of 50 nm. Deposition efficiencies are displayed in Figure 5.

**Figure 6:**
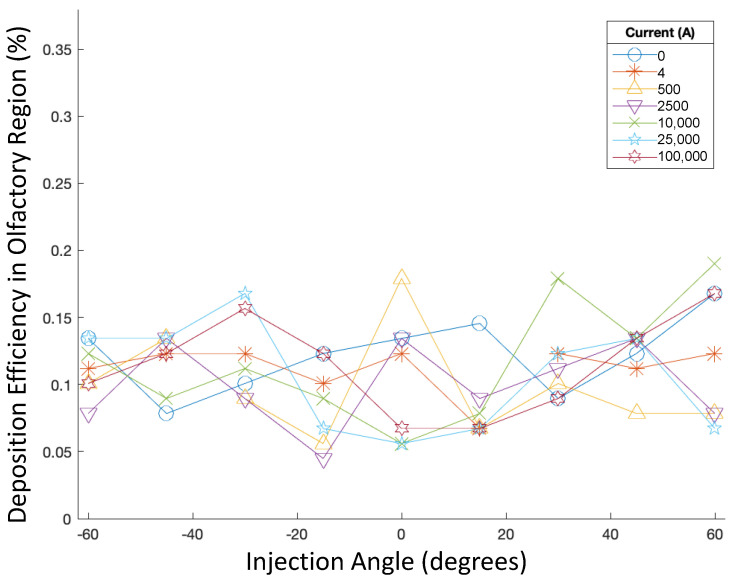
Deposition efficiencies in varying magnetic fields for varying injection angles.

In addition, the injection angle was then varied from $-60^{\circ }$ to $60^{\circ }$ along with the current, while the particle aerodynamic diameter was kept constant at 50 nm, and the injection velocity was kept constant at 5 m/s. Accordingly, the deposition efficiencies are displayed in Figure 6.

All these injections, when injection velocity magnitude and angle were varied, corresponded to extremely low olfactory DE of less than 0.2%. For 50 nm particles, injection velocity magnitude did not have a noticeable effect on olfactory DE. Out of the injections tested for varying injection velocity magnitudes (see Figure 5), the highest DE was achieved when particles were injected at 5 m/s in the magnetic field produced by 500 A of current. Nevertheless, no obvious relationships appear to exist between injection velocity magnitude and particle DE in varying magnetic fields.

Injection angle did not have a noticeable effect on the deposition efficiency of 50 nm particles injected at 5 m/s, as all DEs also remained less than 0.2% (see Figure 6). For each injection angle, modulating the current did not have any significant effects on deposition efficiency either.

**Figure 7:**
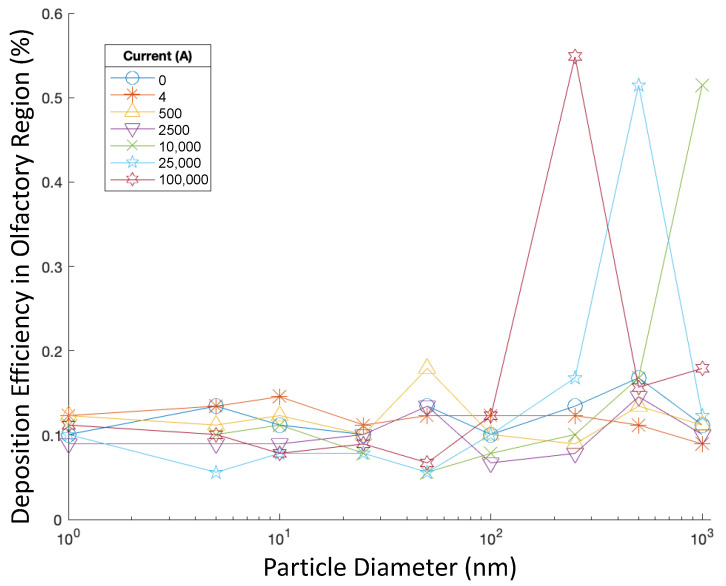
Deposition efficiencies of particles of varying size injected at 5 m/s and in varying magnetic fields.

To further investigate the influence of particle size on the delivery efficiency, olfactory deposition was recorded when injection velocity was constant at 5 m/s, and injections with varying particle aerodynamic diameters were tested in varying magnetic fields. Particles were injected perpendicular to the nasal inlets, and particle aerodynamic diameters ranged from 1 to 1000 nm. Figure 7 shows the DEs for these cases.

When particle aerodynamic diameters were small, changes in diameter did not significantly affect olfactory DE, and magnetic field strength did not have a noticeable effect either. However, for 250 nm particles, the magnetic field generated by 100,000 A of current corresponded to a significant increase in DE, at 4.08 times of the DE when no current was present. Additionally, when 500 nm and 1000 nm particles were injected with magnetic fields generated by currents of 25,000 A and 10,000 A, respectively, the olfactory DE was multiplied by 3.06 and 4.00, respectively, compared with the control cases without current. Using a 2500 A current with 1000 nm particles also corresponded to a rate of olfactory DE multiplied by 3.50 compared with the control case. When nanoparticles with greater aerodynamic diameters and charges, such as particles with diameters of 500 nm and 1000 nm, were paired with stronger magnetic fields (i.e., generated by 100,000 A), the olfactory DE decreased accordingly. It was observed in three-dimensional plots of particle positions that, in these cases, many particles deposited on the front wall of the nasal cavity.

Particles reaching the olfactory region had original positions located near the front of the nasal inlets. Based on the approximate locations of these positions, the initial targeted injection points were selected.

**Figure 8:**
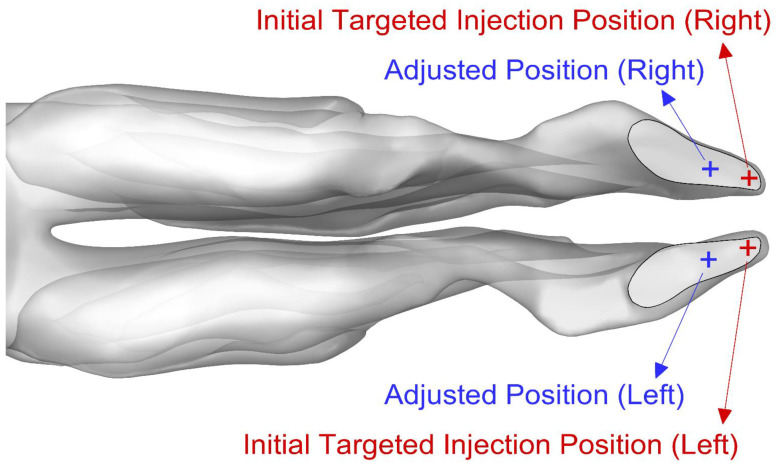
Initial and adjusted positions for targeted injections.

The positions for the adjusted targeted injections were shifted several millimeters back from initial positions. Figure 8 shows the targeted injection positions.

### 3.3. Particle Deposition: Targeted Injections

#### 3.3.1. Initial Targeted Injections

**Figure 9:**
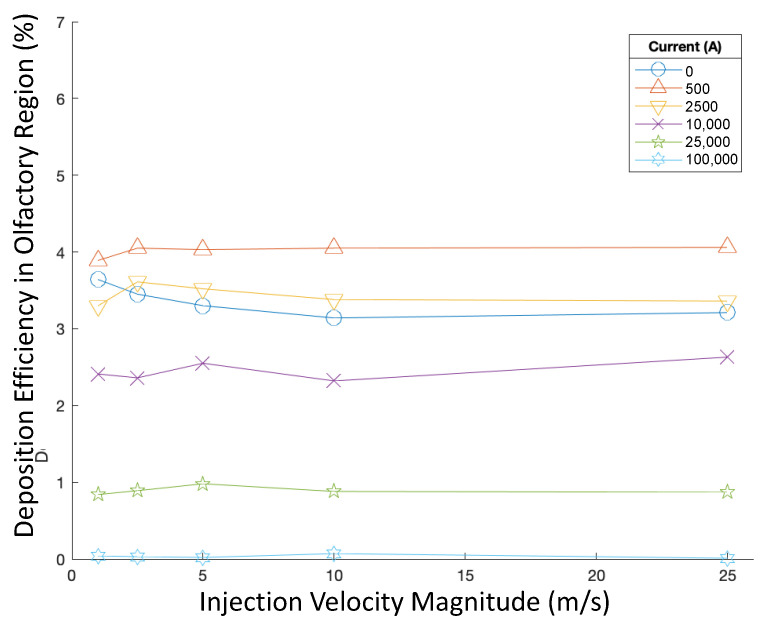
Deposition efficiencies in varying magnetic fields for different injection velocity magnitudes of targeted injections.

Particles were released using spray cone injections from designated positions at each nasal inlet. Olfactory depositions were recorded when the injection velocity magnitude was modulated from 1 m/s to 25 m/s and varying currents, from 0 A to 100,000 A, were run through the wire. All particles had a diameter of 250 nm, and the cone injection’s central axis was angled perpendicular to the boundary surface of the nasal inlet. Figure 9 visualizes and compares the DEs in the olfactory region for these cases.

**Figure 10:**
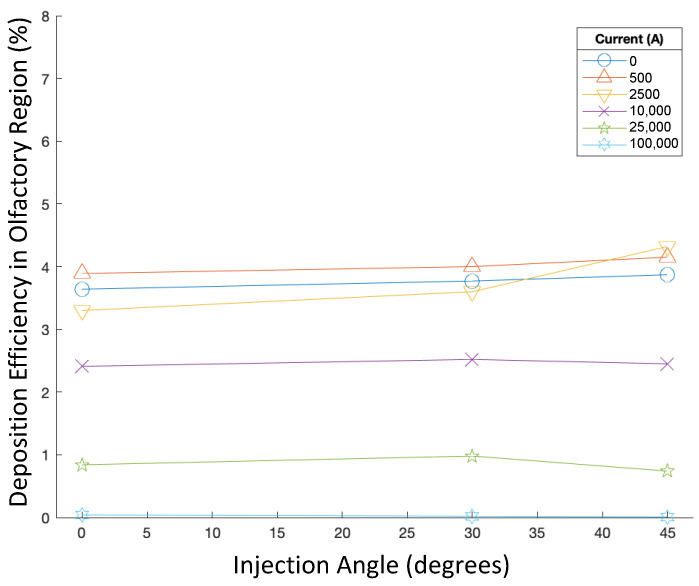
Deposition efficiencies in varying magnetic fields for different injection angles of targeted injections.

Deposition efficiency was also recorded when the injection angle was modulated from $0^{\circ }$ to $45^{\circ }$. The particle diameter and injection velocity magnitude were held constant at 250 nm and 1 m/s, respectively (see Figure 10 for the DEs of these cases).

Injection velocity magnitude had no significant effect on the deposition efficiency for 250 nm particles. However, varying magnetic field strengths did noticeably influence olfactory deposition. Out of the currents tested, 500 A corresponded to the highest deposition efficiency for varying injection velocity magnitudes (see Figure 9), while further increasing the current corresponded to noticeable decreases in deposition efficiency. With currents of 50,000 A and 100,000 A, nearly no particles deposited in the olfactory region.

Injecting particles from the targeted release point corresponded to significant increases in DE, compared with uniform injections. Most uniform injections achieved less than 0.2% DE, with the highest DE being less than 0.6%. With the targeted release strategy, olfactory DEs of approximately 4% were achieved with 500 A of current.

For targeted injections, injection angle also did not have a significant effect on the DE of 250 nm particles injected at 1 m/s (see Figure 10). For each injection angle out of these cases, increasing the current generally resulted in lower DE, while the highest DE achieved in these cases with a varying injection angle was slightly higher than 4% when the current was 2500 A and the injection angle was 45${}^{\circ }$.

**Figure 11:**
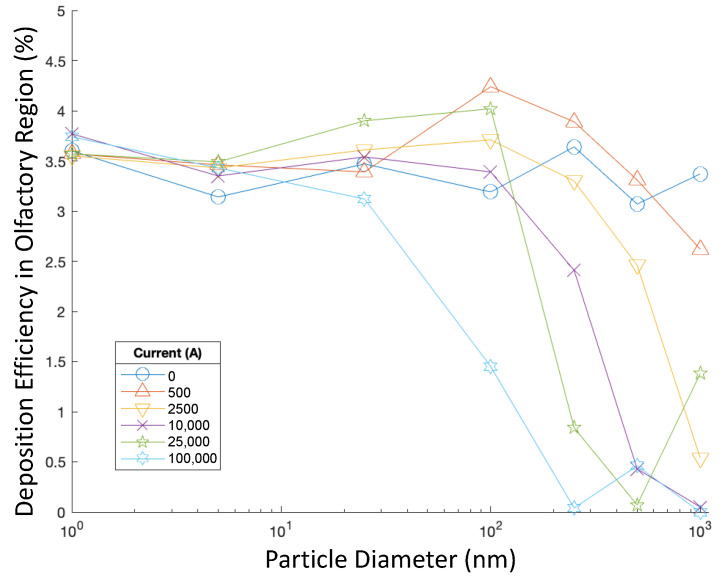
Olfactory deposition efficiencies of particles of varying size injected at 1 m/s in varying magnetic fields using targeted injections.

Olfactory DE was also recorded for targeted injections with particles of varying sizes, from 1 nm in diameter to 1000 nm, injected at a constant velocity magnitude of 1 m/s perpendicular to the inlet boundaries and in varying magnetic fields. Figure 11 shows the DEs for these cases.

When no external magnetic field was present, particle diameter did not significantly influence the DE in the olfactory region when the targeted release strategy was used. However, when greater currents were run through the wire, increasing particle diameter corresponded with decreased DE. When the particle diameter was small, the current did not have a significant effect on deposition. However, as the particle size increased, the differences in DE for differing currents began to increase. A greater particle diameter appears to correlate with more significant decreases in DE when greater currents were applied.

Out of all cases tested, the highest DE of 4.24% was achieved when 100 nm particles were injected using a targeted injection and the current was at 500 A. DEs when targeted injections were applied were generally much greater than uniform injections, excluding cases with very high currents. For cases with higher currents, it was also observed in plots of DEs that many particles deposited in the front of the nasal cavity before the olfactory region.

#### 3.3.2. Adjusted Targeted Injections

**Figure 12:**
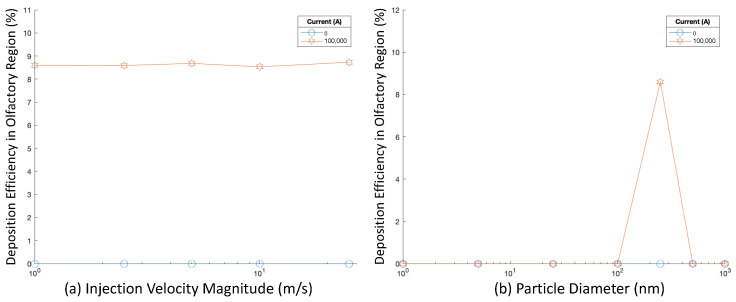
Deposition efficiency with varying magnetic fields for varying injection velocity magnitudes and diameters for targeted injections: (**a**) Deposition efficiency vs. injection velocity, and (**b**) Deposition efficiency vs. injection angle.

Adjusted positions of release for targeted injections were also tested. The same trials were conducted as with initial targeted injections, with only the position of injection differing. Figure 12 shows deposition efficiencies for varying injection velocity magnitudes and particle diameters.

When particles were released from the adjusted injection position, injection velocity magnitude did not significantly affect deposition efficiency. When no magnetic fields were applied, no particles reached the olfactory region. However, when 100,000 A of current was applied, the DE was above 8%.

Particle diameter influenced particle delivery efficiency. When no current was present, olfactory deposition remained at zero. However, when 100,000 A of current was applied and the particle diameter was at 250 nm, a DE above 8% was achieved—the highest out of any of the diameters tested.

**Figure 13:**
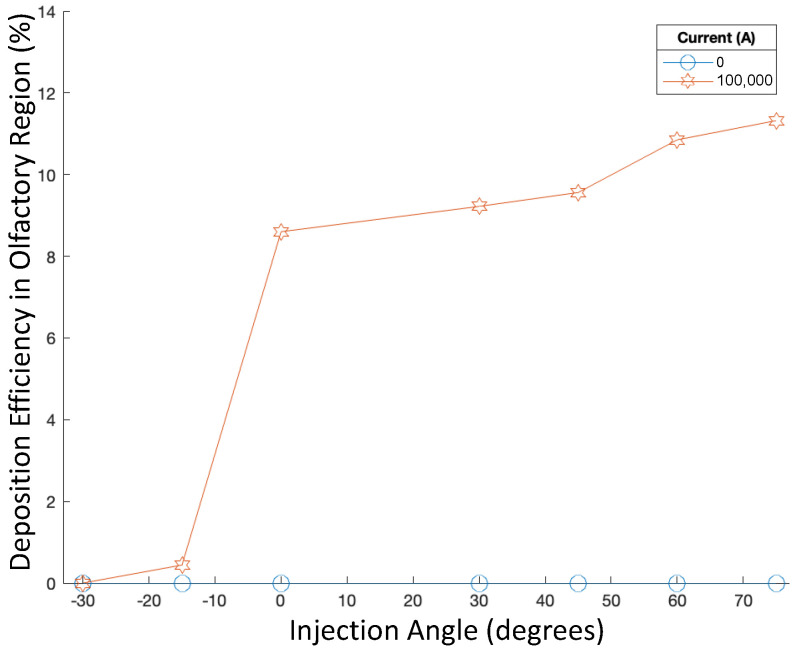
Deposition efficiency in varying magnetic fields for varying injection angles for targeted injections.

**Figure 14:**
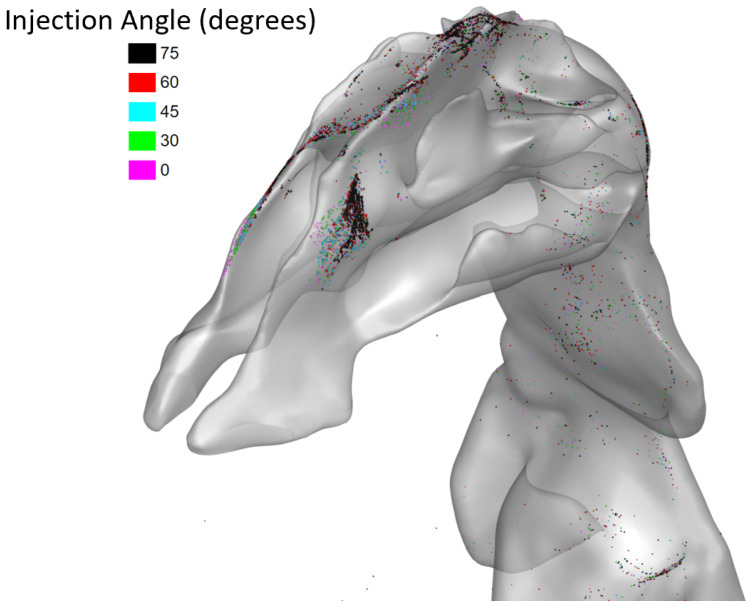
Particle deposition positions with varying injection angles for 100,000 A current.

With the adjusted position for targeted injections, the injection angle was modulated from $-30^{\circ }$ to $75^{\circ }$ while the particle diameter and injection velocity were held constant at 250 nm and 1 m/s. The recorded deposition efficiencies are displayed in Figure 13. In Figure 14, the deposition of particles in the nasal cavity and olfactory region is displayed.

For angles from $-30^{\circ }$ to $75^{\circ }$, there seems to exist a positive correlation in Figure 13 between injection angle and deposition efficiency, specifically for 250 nm particles injected from the adjusted position of targeted injections when the current was at 100,000 A. When no current was present, deposition remained at zero for all angles. However, for large injection angles, deposition efficiency reached up to 11.32%. This correlation is visible in Figure 14. Greater injection angles (i.e., angled more toward the back of the nasal cavity) corresponded to more deposition in the olfactory region.

### 3.4. Drug Diffusion through Mucus

Based on the DE found from CFD simulations, the percentage of injected drug absorbed at the olfactory epithelium was also calculated.

**Figure 15:**
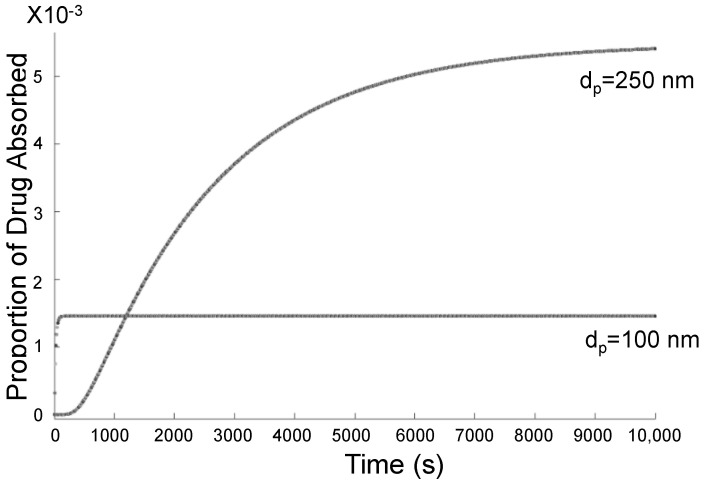
Proportion of total injected drug absorbed through olfactory epithelium over time.

Figure 15 displays the proportion of the total injected drug that was absorbed at the olfactory region over time for two injections. For example, if the current was at 100,000 A and injections were uniform, there were more 250 nm particles deposited in the olfactory region compared with 100 nm particles, but for the smaller 100 nm particles, drug absorption occurred at a higher rate.

## 4. Discussion

### 4.1. Airflow Dynamics in the Nasal Cavity

Airflow was a prominent factor for particle trajectories, especially for nanoparticles. Due to complex local airflow velocity distributions in the nasal cavity, the position a particle was injected at was important for determining its trajectory and whether it reached the olfactory region. With the small size of the nano-/micro- particles, they tended to follow the nasal airflow well with small Stokes numbers [42]. Particles injected at the front of the nasal inlets often traveled along airflow streamlines to reach upper areas of the nasal cavity, such as the olfactory region. Particles injected in the middle of the nasal inlets traveled through the middle regions of the nasal cavity, and particles injected at the back of the middle cavity traveled near the bottom of the nasal cavity. The trajectories of these small particles indeed conformed well with paths of nasal airflow. Many particle trajectories even entered the olfactory region but followed the airflow to exit the region.

The conformity of particle trajectories to airflow streamlines, in addition to drag, offers a reason for the often negligible influence of injection velocity magnitude on deposition efficiency. For uniform injections and initial targeted injections, injection velocity did not play a significant role. Because drag force relates to the velocity difference between the airflow and the particle, and due to the strong effect of drag on small particles, of which the surface area to volume ratio is larger than that of particles with large diameters, injecting particles at higher velocities leads to stronger opposing drag forces initially. The velocity difference between small particles and airflow will reach zero quickly. Therefore, the initial injection velocity of small particles, especially nanoparticles, has a negligible influence on their transport and deposition in the nasal cavity.

Regions of air recirculation in front of the olfactory region, revealed by visualizations of airflow velocity, also influenced particle trajectories and deposition. Bates et al. [43] also observed similar airflow recirculation patterns in the right side of the nasal cavity. As air flowed through the inlets, it accelerated as it passed over angled portions of the inner walls, which was evident in the high airflow velocities in the front of the nasal cavity. This resulted in flow separation in the top of the nasal cavity in front of the olfactory region, causing air to flow back and form a recirculation zone. During the 0.5 s period simulated, a fraction of particles remained in the recirculation and did not deposit. This further shows the significant influence of airflow on particles.

### 4.2. External Magnetic Field and Particle Trajectories

CFD simulations with varying levels of current modeled effects of magnetic forces, generated by the current-carrying wire, on particles. Control cases corresponded to very low olfactory deposition rates. Even with magnetic forces, deposition efficiency remained below 0.2% in many cases. However, for larger nanoparticles with diameters of 250 nm, 500 nm, and 1000 nm, certain levels of current led to over three-fold increases in deposition efficiency. Thus, external magnetic fields have the potential to improve olfactory delivery efficiency. Moreover, there may exist a relationship between particle diameter and the magnetic field strength needed to enhance efficiency.

For smaller nanoparticles of 100 nm or less in diameter, there were no significant influences on deposition efficiency for the tested levels of current. Based on the assumptions for particle charges, the magnetic force per mass exerted on each particle was equal, regardless of particle size. This lack of response may be explained by drag forces. Smaller nanoparticles have a greater ratio of surface area to volume, and drag directly relates to an object’s area. Thus, smaller particles experience a greater force per mass due to drag [44]. Thus, particles with smaller diameters experienced greater drag force per mass. This offers an explanation of the lack of influence of the tested magnetic fields on smaller nanoparticles. Specifically, all particles experienced equal magnetic forces per mass, but smaller particles experienced greater drag forces per mass.

This principle of drag provides a reason for certain levels of current significantly improving deposition efficiency for particles of a certain diameter, but not of other diameters. For 250 nm, 500 nm, and 1000 nm particles, it was observed in uniform injection cases that larger particles corresponded to lower levels of current necessary to enhance deposition efficiency. Since the drag force is inversely proportional to $d_{p}^{2}$, larger particles experienced less drag force per mass. Thus, for larger particles, weaker magnetic fields were needed for the magnetic force exerted on a particle to overcome drag.

Along with the fact that less current was needed to redirect larger nanoparticles, higher levels of current also corresponded to lower deposition efficiency for some cases with larger nanoparticles. This was due to many particles traveling up the front of the nasal cavity being redirected by the magnetic force to deposit on the front walls of the nasal cavity. This was evident in cases with larger nanoparticles when the initial targeted injection was used, as many 250 nm, 500 nm, and 1000 nm particles were drawn toward the front walls of the nasal cavity by the magnetic field. Similarly, 500 nm and 1000 nm particles had low deposition efficiencies when 100,000 A of current was applied, as they deposited before reaching the olfactory region.

The relation between particle size and magnetic force per mass needed to overcome drag forces suggests a balance that can be achieved with particle size and current in order to successfully redirect particle trajectories but avoid magnetic forces that lead particles to deposit on the front walls of the nasal cavity. The position of injection and airflow velocity also influenced the trajectories of the particle and the magnitude and direction of drag. Thus, drag forces are not constant. However, it can be roughly approximated that the magnetic force per mass needed to overcome drag force and further redirect the particle is also inversely proportional to $d_{p}^{2}$. For instance, 100,000 A of current significantly enhanced delivery efficiency for uniform injections of 250 nm particles. Doubling the diameter to 500 nm, 100,000 A of current led to low deposition, but 25,000 A of current resulted in significantly increased deposition efficiency. Doubling the diameter to 1000 nm, 25,000 A of current resulted in low deposition, but a lower current of 10,000 A resulted in enhanced efficiency. While 10,000 A is not exactly 0.25 of 25,000 A, it is possible that some current less than 10,000 A leads to even higher delivery efficiency for 1000 nm particles.

### 4.3. Targeted Injections

The positions for targeted spray cone injections were selected based on approximate starting positions of particles in uniform injections that reached the olfactory region. When lower levels of current were applied, deposition efficiency significantly increased when targeted injections were used. When 500 A of current was applied, deposition efficiencies above 4% were achieved—up to an 20-fold increase from the majority of uniform injections tested. With no magnetic field, efficiencies reached 3% to 4%, which was still much higher than deposition efficiencies for uniform injections. Thus, targeted injections led to significant increases in deposition efficiency.

The airflow streamlines offer an explanation for such improvements. Air entering the nasal cavity at the front of the nasal inlets traveled along the front of the nasal cavity, near the front walls. Some of these airflow streamlines reached the top of the nasal cavity. Particles injected at the front of the inlets and traveling along these streamlines were more likely to land in the olfactory region.

The targeted injection at the very front of the nasal cavity enhanced deposition efficiency. However, it is important to consider that, realistically, nasal sprays do not inject particles in a perfectly straight line, but often inject particles in a cone-shaped plume [45]. Spray cone injections were used in this study, and with the spread of injected particles, as well as the constricted region at the front of the nasal inlet where particles are injected, many particles ended up depositing in the nasal vestibules or front walls of the nasal cavity. While increasing the injection angle led to less particle deposition in the vestibules, it did not significantly improve deposition efficiency, as the angled injection may have caused more particles to follow airflow streamlines below the olfactory region.

Injecting particles at the adjusted positions, further back on the nasal inlets, led to no olfactory deposition when no magnetic fields were applied. This suggests that, when only injection position was considered without magnetic fields, using the particle release map technique and targeted injections led to increased olfactory deposition.

Nevertheless, when magnetic fields were introduced, especially stronger magnetic fields, deposition efficiency decreased in some cases. For small nanoparticles, with diameters such as 1 nm and 5 nm, increasing magnetic field strength did not greatly influence deposition efficiency. However, for larger particles, increases in magnetic field strength resulted in increasingly drastic reductions in deposition efficiency, with no 1000 nm particles reaching the olfactory region when 100,000 A of current was applied. Since particles were released at the front of the nasal inlet, many of them likely traveled close to the front walls of the nasal cavity along the airflow in the front of the nasal cavity. Thus, increasing current led many particles to deposit on the front walls of the nasal cavity.

While targeted injections alone did enhance deposition efficiency, the large number of particles deposited in the vestibules and on the front wall were not ideal. Furthermore, when magnetic forces were applied to draw particles closer to the front of the nasal cavity for higher likelihood of depositing in the olfactory region, many particles deposited on the front walls before reaching the olfactory region. Thus, only using targeted injections still comes with limitations.

### 4.4. Integration of Magnetic Fields and Targeted Injections

Initial targeted injections resulted in many particles depositing in the vestibules and front walls of the nasal cavity. When magnetic fields were combined with the initial targeted injections, increasing magnetic field strength had negative effects on deposition efficiency. However, the influence of magnetic fields on particle deposition was also dependent on the injection position of the particles.

When targeted injection positions were adjusted by several millimeters toward the middle of the nasal inlets, no particles reached the olfactory region when no current was applied. However, by injecting 250 nm particles from the adjusted targeted injection position and applying 100,000 A of current, significant increases in deposition efficiency were achieved. Compared with the initial targeted injections, this integration of magnetic force with the adjusted targeted injections resulted in deposition efficiencies that were two to nearly three times higher.

By shifting the targeted injection position back from the very front of the nasal inlets, less particle deposition in the vestibules occurred, allowing more particles to be injected deeper into the nasal cavity. Additionally, this adjusted injection position meant that more particles traveled slightly further away from the front walls of the nasal cavity. This allowed the magnetic field to draw more particles to the olfactory region, rather than causing them to deposit too early. In addition, deposition efficiency increased as the injection angle increased toward $75^{\circ }$. This could also be explained by angled injections releasing more particles at a farther distance from the front walls of the nasal cavity, allowing the magnetic force to guide more particles to the olfactory region instead of to the front walls.

Therefore, while using the initial targeted injection alone led to greatly increased deposition efficiency in the olfactory region, further adjusting the targeted injection position and angle to account for the magnetic force and spread of the spray cone led to even greater deposition efficiencies.

### 4.5. Drug Diffusion and Absorption

A larger particle size corresponds to a lower diffusion coefficient, meaning slower diffusion through the mucus layer covering the olfactory epithelium. On the other hand, larger particles experience less drag force, meaning weaker magnetic forces can more easily draw more particles toward the olfactory region. In some cases, there exists a trade-off between deposition efficiency and the time needed for the drug to diffuse through the mucus layer before it is actually absorbed through the olfactory epithelium. In other words, it may be easier to achieve higher deposition efficiency using magnetic fields on larger nanoparticles, but these particles would take longer to be absorbed compared to smaller nanoparticles. Depending on the intention of medical treatment, whether a small amount of a drug must be absorbed quickly, or a larger amount must be absorbed gradually, specific particle diameters would be more suitable.

It is not enough to only consider deposition efficiency, as factors playing a role in efficiency also influence other steps in the drug delivery process. Drug diffusion through the mucus layer is a factor that must be weighed when configuring nasal drug delivery. The amount of drug absorbed, as well as the absorption rate, depends on particle diameter, as well as deposition efficiency, which relates to the particle diameter, magnetic force, and injection position. Calibration between the components is necessary to achieve desired outcomes.

### 4.6. Implications

These findings display the roles that fluid flow, particle diameter, magnetic forces, and targeted injections play in drug delivery efficiency. Moreover, insights are revealed regarding the meaningful connections between these factors, which allow for better integration to achieve enhanced delivery efficiency. While initial positions for targeted injections allowed for spray cone injections that led to improvements in olfactory delivery efficiency, more factors must be considered when combining targeted injections and magnetic fields to achieve even higher efficiencies.

This study elucidates the relationships between such aspects of drug delivery and reveals that they do not independently affect drug deposition efficiency but are interconnected with other variables. Ideal magnetic fields that improve efficiency relate to drag forces, which are connected to particle diameter. A suitable magnetic field strength also relates to the position and angle of targeted injections. Furthermore, the particle diameter also affects its diffusion rate through mucus.

In addition, realistic factors, such as the inhalation waveform and spray cone injections, further add to the comprehensiveness of insight provided by the simulations. For example, applying the spray cone injection led to results suggesting that the initial targeted injection position may have caused many particles to deposit in the vestibules. This explains why the adjusted targeted injection, in use with the magnetic field, achieved over twice the deposition efficiency of the initial targeted injection and over 65 times the efficiency of the uniform injection of 250 nm particles with no magnetic field.

The findings of this study can inform designers of nasal drug delivery systems regarding how to effectively integrate magnetic fields, targeted injections, and nasal sprays with specific properties to achieve more efficient olfactory drug delivery. The many factors considered, including drug diffusion through nasal mucus and absorption at the olfactory epithelium, capture a broad view of drug delivery. This can lead to more complex analyses of drug delivery models that are even more comprehensive and physiologically realistic.

### 4.7. Limitations and Future Work

Only an infinite linear wire was tested in this study. Other configurations of magnetic targeting systems may reveal alternative ways to further improve olfactory drug delivery [46,47,48]. Such systems can be further examined in future work.

Particles remaining in the recirculation region also present a limitation of this study. If simulations were extended to include airflow through the end of inhalation and into the exhalation period, more insight would be gained on where the particles in the recirculation region might deposit. This suggests future work to simulate full breathing cycles, multiple breathing cycles, or an aspect of randomness in breathing patterns.

The inhalation flow rate used in this study is based on a realistic breathing waveform. However, there is a possibility of variation in the inhalation rate, which may influence drug deposition. Future work with various inhalation waveforms may provide a more comprehensive understanding of olfactory drug delivery.

Magnetic fields simulated in this study may not be able to be directly created in physical experiments, due to unfeasible currents and magnetic field strengths that could be unsafe for humans. However, the understanding gained still reveals the levels of magnetic forces that are suitable to enhance efficiency, which can be applied in future work to develop and improve drug targeting using externally generated forces.

It is also worth mentioning that the nasal cavity geometry selected for this study does not contain sinuses [22]. To increase the realism of the numerical simulation, influences of the sinuses on airflow and drug particle trajectories as well as the targeted delivery efficiency will be investigated in the future.

## 5. Conclusions

In this study, the delivery of charged nasal spray nanoparticles with magnetic drug targeting was simulated using computational fluid dynamics, with the RANS model for nasal airflow and the discrete phase model for particle trajectories. The magnetic field generated by a current-carrying wire was modeled to determine its influence on particle deposition. Experiments examined the effects of parameters—namely particle size, the velocity magnitude, angle, and position of injection, and magnetic field strength—on drug delivery efficiency to the olfactory region and drug diffusion through the mucus layer, with the aim of determining how the olfactory delivery efficiency of nanoparticles can be enhanced. Findings support the idea that, due to drag forces, the combination of particle diameter, injection position, and magnetic field strength is the deciding factor in olfactory delivery efficiency. The inverse relationship between particle size and ideal magnetic field strength that enhances efficiency, as well as ideal injection positions based on magnetic field strength, suggests how to integrate the three factors to achieve the best efficiency. With a 100,000 A wire current and adjusted positions for targeted spray injections, the delivery efficiency of 250 nm nanoparticles was enhanced by over 65-fold compared with control cases with uniform injections and no magnetic field. This study provides insight that can inform physical drug delivery experiments on combinations of particle size, injection position, and external guiding forces that significantly increase efficiency, as well as future experiments and clinical studies that integrate even more factors of nasal drug delivery.

## Figures and Tables

**Table 1:** Coefficients of the sinusoidal series describing the nasal breathing waveform (see Equation (6)).

i	$a_{i}$	$b_{i}$	$c_{i}$
1	263.7	0.6369	3.015
2	330.7	0.1254	−3.727
3	73.19	4.86	−0.7755
4	48.58	7.822	−0.7588
5	16.06	13.33	0.03013
6	253.6	2.011	−0.4039
7	43.69	10.85	−1.085

## Data Availability

The data that support the findings of this study are available from the corresponding author upon reasonable request.

## Abbreviations

The following abbreviations are used in this manuscript:

BBB	Blood–brain barrier
CAD	Computer-aided design
CFD	Computational fluid dynamics
CNS	Central nervous system
DE	Deposition efficiency
DPM	Discrete phase model
HPCC	High Performance Computing Center
LES	Large eddy simulation
OSU	Oklahoma State University
RANS	Reynolds-averaged Navier–Stokes
SST	Shear–stress transport
UDF	User-defined function
